# Mechanical characterization and torsional buckling of pediatric cardiovascular materials

**DOI:** 10.1007/s10237-023-01809-z

**Published:** 2024-02-15

**Authors:** Samir Donmazov, Senol Piskin, Tansu Gölcez, Demet Kul, Ahmet Arnaz, Kerem Pekkan

**Affiliations:** 1 Department of Mathematics, University of Kentucky, Kentucky, 40506 USA; 2 Department of Mechanical Engineering, Istinye University, Istanbul, 34010 Turkey; 3 Department of Bio-Medical Science and Engineering, Koc University, Istanbul, Turkey; 4 Department of Cellular and Molecular Medicine, Koc University, Istanbul, Turkey; 5 Department of Cardiovascular Surgery, School of Medicine, Acibadem Mehmet Ali Aydinlar University, Istanbul, Turkey; 6 Department of Mechanical Engineering, Koc University, Sariyer, Istanbul Turkey

**Keywords:** Congenital heart disease, Surgical patch design, Torsion, Vascular mechanics, Fontan, Conduit, Material characteristics, Nonlinear optimization, Tissue engineered patches, PTFE, Pericardium

## Abstract

In complex cardiovascular surgical reconstructions, conduit materials that avoid possible large-scale structural deformations should be considered. A fundamental mode of mechanical complication is torsional buckling which occurs at the anastomosis site due to the mechanical instability, leading surgical conduit/patch surface deformation. The objective of this study is to investigate the torsional buckling behavior of commonly used materials and to develop a practical method for estimating the critical buckling rotation angle under physiological intramural vessel pressures. For this task, mechanical tests of four clinically approved materials, expanded polytetrafluoroethylene (ePTFE), Dacron, porcine and bovine pericardia, commonly used in pediatric cardiovascular surgeries, are conducted (*n* = 6). Torsional buckling initiation tests with *n* = 4 for the baseline case (*L* = 7.5 cm) and *n* = 3 for the validation of ePTFE (*L* = 15 cm) and Dacron (*L* = 15 cm and *L* = 25 cm) for each are also conducted at low venous pressures. A practical predictive formulation for the buckling potential is proposed using experimental observations and available theory. The relationship between the critical buckling rotation angle and the lumen pressure is determined by balancing the circumferential component of the compressive principal stress with the shear stress generated by the modified critical buckling torque, where the modified critical buckling torque depends linearly on the lumen pressure. While the proposed technique successfully predicted the critical rotation angle values lying within two standard deviations of the mean in the baseline case for all four materials at all lumen pressures, it could reliably predict the critical buckling rotation angles for ePTFE and Dacron samples of length 15 cm with maximum relative errors of 31% and 38%, respectively, in the validation phase. However, the validation of the performance of the technique demonstrated lower accuracy for Dacron samples of length 25 cm at higher pressure levels of 12 mmHg and 15 mmHg. Applicable to all surgical materials, this formulation enables surgeons to assess the torsional buckling potential of vascular conduits noninvasively. Bovine pericardium has been found to exhibit the highest stability, while Dacron (the lowest) and porcine pericardium have been identified as the least stable with the (unitless) torsional buckling resistance constants, 43,800, 12,300 and 14,000, respectively. There was no significant difference between ePTFE and Dacron, and between porcine and bovine pericardia. However, both porcine and bovine pericardia were found to be statistically different from ePTFE and Dacron individually (*p* < 0.0001). ePTFE exhibited highly nonlinear behavior across the entire strain range [0, 0.1] (or 10% elongation). The significant differences among the surgical materials reported here require special care in conduit construction and anastomosis design.

## Introduction

Congenital heart defects are associated with a spectrum of structural malformations including atrial or ventricle septal defects (Marelli et al. 2007), venous/arterial malformations and missing or flipped great vessels and ventricles. These defects are palliatively repaired through complex multi-stage reconstructive surgeries (Ball et al. 2016), wherein artificial conduits or vascular patches are anastomosed to the native tissue of the failed ventricular or vascular region (Lashkarinia et al. 2018). For example, in a Fontan reconstruction a circular artificial baffle type conduit is employed to connect the inferior vena cava to the right pulmonary artery (Fontan and Baudet 1971).Likewise, the first-stage neonatal cardiovascular interventions also involve end-to-side anastomosis of small diameter shunts to direct flow from the ventricle or aorta to the main pulmonary artery (Norwood et al. 1981). Due to a variety of materials involved and complex anatomies, the biomechanics of the pediatric surgical anastomosis site is complex and received limited attention in the literature. Most importantly, the complex 3D vessel shapes are likely to result in surface wrinkles at the anastomosis region and can reduce postoperative hemodynamic performance. The severity of large-scale surface deformations depends on the level of intramural pressure and governed primarily due to the torsional deformation introduced between the two anastomosed vessels. Therefore, the main motivation of the present article is to investigate the torsional buckling characteristics of end-to-side anastomosis sites considering the variety of pediatric surgical materials that are alternatively available for the same surgical repair operation.

Torsional buckling in arteries and veins, resulting from vascular instability, is likely to play a significant role in the development of postoperative pathologies influencing graft patency. Han et al. introduced a biomechanical model for the arterial buckling and established a mathematical framework to analyze the effect of the internal pressure and torsion on the mechanics of arteries and veins using both linear and nonlinear models (Han 2007, 2009; Lee and Han 2010; Martinez et al. 2010; Lee et al. 2012; Martinez and Han 2012; Garcia et al. 2013; Han et al. 2013; Hayman et al. 2013; Liu et al. 2014). Their findings indicated that vascular buckling could occur due to the reduced axial strain, increased blood pressure and the characteristics of the flexible vascular walls, considering the changes in critical buckling pressure. Han and his collagenous also conducted experimental investigations on the buckling behavior of arteries under torsion, aiming to determine the critical buckling torque, the critical buckling twist angle and buckling shape (Garcia et al. 2013). They observed that the critical buckling rotation angle and twist angle at specific axial stretch ratios exhibited a biphasic response with increasing lumen pressure. To the best of our knowledge, in addition to studies by Han et al. mentioned above, there is only one more study that focuses on *in vitro* experiments investigating the torsional buckling of vascular conduits performed by our group (Oguz et al. 2017), where Fontan conduit materials were incorporated into a mock-up total cavopulmonary connection (TCPC) circuit, mimicking mechanically failed inferior vena cava (IVC) anastomosis morphologies. This study showed that large-scale surface deformations observed in torsional buckling can lead to an order of magnitude increase in blood flow resistance. In the present manuscript, focusing structural mechanics, we have expanded the experimental arm by investigating the critical (buckling) rotation angle values for the four commonly used pediatric surgical materials. This large-scale experimental campaign allowed us to explore a possible theoretical formulation that can be generalized to any surgical conduit. A generalized buckling formulation is desired, specifically for pediatric cardiovascular applications where new and more advanced surgical materials are routinely becoming available for surgeon’s use. As such, in this study, a predictive tool is proposed that would incorporate the mechanical factors and geometrical differences of emerging surgical materials having different buckling characteristics.

Another aim of the present study is to develop a generalized formulation that can be incorporated in computational biomechanical modeling and can be expanded to broader soft tissue applications. Therefore, the strain energy density function is adopted here to describe and reproduce complex mechanical response of the vascular tissue (Holzapfel et al. 2000; Gasser et al. 2006; Peña et al. 2010). Among many studies, for example, this approach also applicable to study the mechanics of disease states, including the ageing of the human aorta (Zulliger and Stergiopulos 2007). Contemporary seminal studies by Chuong and Fung (1984), Fung (1990) and his successors such as Holzapfel, Gasser, Ogden (2000) and Humphrey (2002) proposed constitutive relations that describe the mechanical response vascular tissue (Peña et al. 2010). For instance, the transmural stress distribution, which strongly influences the mechanobiological processes occurring in the vascular tissue, can be computed (Humphrey 2002). However, most studies in the literature did not focus on pediatric surgical materials and target high operating pressure arterial tissue. The present article aims to address these gaps by focusing pediatric disease states and study the response at lower venous pressure levels.

## Methods

### Sample preparation and mechanical tests

**Fig. 1:**
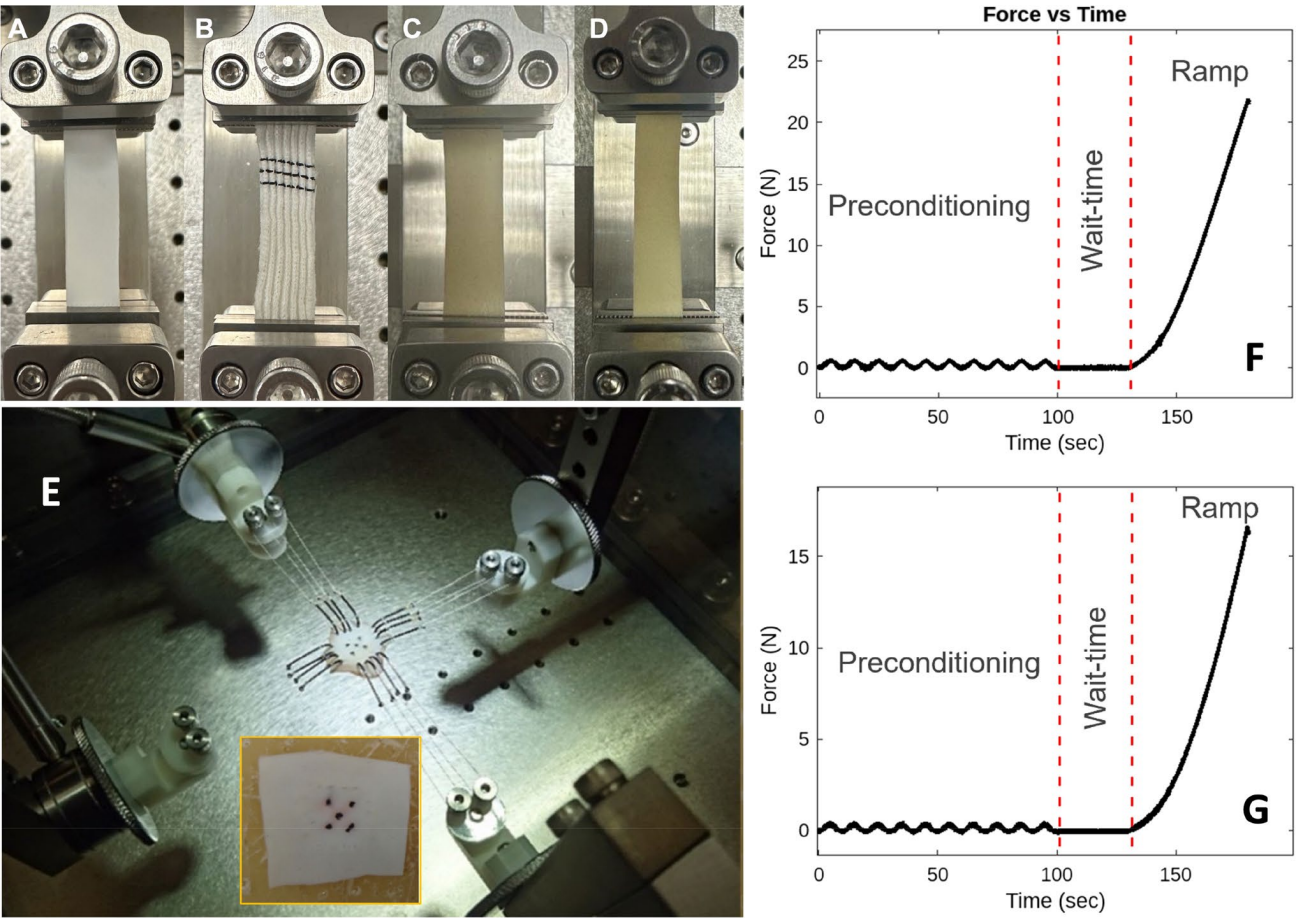
Vessel segments placed on the uniaxial test setup clamps, **A** ePTFE, **B** Dacron, **C** bovine pericardium and **D** porcine pericardium. **E** Biaxial tensile test setup with the porcine pericardium specimen placed for the experimental protocol, and the porcine pericardium specimen after being used in the biaxial tensile test. The force–time data obtained from uniaxial tensile tests of **F** ePTFE and **G** bovine pericardium, both stretched in the axial direction

Sections of material specimens were extracted from the commercially available 20-mm-diameter circular conduits specifically produced for pediatric cardiovascular surgeries. As in routine surgical use, pericardium samples were preserved in glutaraldehyde solution following the standard surgical protocol and promptly subjected to testing. For uniaxial tests, specimens were prepared for each material in both circumferential and axial orientations, with a sample dimensions of 1 × 5 cm (Fig. 1A–D). In the case of biaxial testing, square specimens measuring 2 × 2 cm were marked with a permanent marker to facilitate tracking with the overhead camera. These specimens were then connected to the test system (BOSE planar biaxial test system, BOSE Inc., MA, USA) using multiple small connecting hooks (Fig. 1E).

#### Uniaxial tensile tests

The uniaxial tensile tests were performed on both axial and circumferential specimens of ePTFE (expanded polytetrafluoroethylene) (Goretex, USA), Dacron (Jotec, DE), and porcine (Biointegral, CA) and bovine pericardia (Edwards Lifesciences, CA, USA), with *n* = 3 for each direction. All samples were tested under two conditions: preconditioning and ramp, respectively. A sine wave was applied for 10 cycles with a frequency rate of 0.01 mm/sec during the preconditioning state. At the end of the preconditioning test, all samples were left without applying a load for 30 s. Subsequently, a force was applied at a speed of 0.1 mm/sec to create a displacement of 5 mm for the ramp test.

#### Biaxial tensile tests

The samples were aligned in both axes and attached to the linear motors using hooks (Fig. 1E). Preconditioning cycles were initiated after eliminating any slack and calibrating the load cells in both axes. A total of 100 loading and unloading cycles were applied to the specimens at a frequency of 0.1 Hz. Each cycle consisted of 50 data points, with 25 data points recorded during sample loading and 25 during unloading. The specimens underwent a total displacement of 4 mm in each direction after 40% stretching. The data from the first 50 cycles were excluded for preconditioning and not included in the subsequent analysis. This testing procedure was performed for ePTFE, Dacron and porcine pericardium.

#### Torsional buckling experiments

**Fig. 2:**
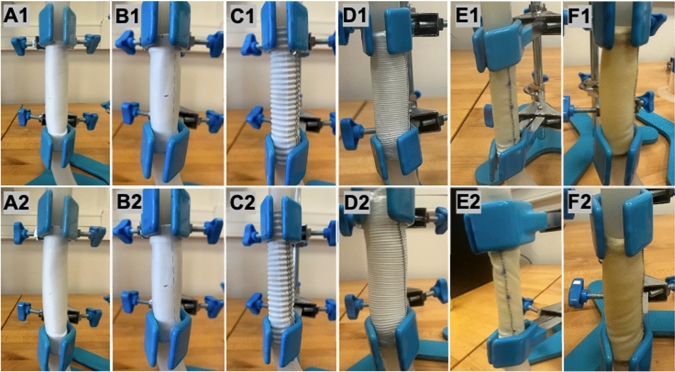
The configuration with no torque (**A1**, **B1**, **C1**, **D1**, **E1** and **F1**) and the moment when the initial buckling first becomes barely visible just before rapid collapse (**A2**, **B2**, **C2**, **D2**, **E2** and **F2**) for each sample (**A** and **B** ePTFE, **C** and **D** Dacron, **E** Bovine Pericard, F:Porcine Pericard) are shown. (**A** GORE-TEX, **B** B.Braun, **C** Getinge, **D** Jotec, **E** Edwards Lifesciences, **F** BioIntegral)

To measure the critical buckling rotation angle, tubular ePTFE and Dacron (PET-polyethylene terephthalate) vascular grafts, as well as porcine and bovine pericardial patches, were procured with a diameter of 20 mm and lengths of 7.5 cm and 5 cm, resulting in a sample size of *n* = 4 for each material. Additionally, tubular ePTFE and Dacron graft samples were obtained at a length of 15 cm, with a sample size of *n* = 3 for each. Dacron samples at a length of 25 cm were also acquired, with *n* = 3 samples having the same diameter and thickness values as the previous samples. Samples in ePTFE and Dacron include two different commercial brands (GORE-TEX and B.Braun for ePTFE, and Getinge and Jotec for Dacron) as shown in Fig. 2. All ePTFE samples tested were of the same thickness, 0.9 mm; Dacron samples had 0.65 mm, except for one with 0.6 mm; porcine pericardium samples had 0.63 mm and bovine pericardium samples had 0.72 mm thickness, measured via optical coherence tomography. The experimental setup includes a short rigid tubing of the same internal graft diameter, which was affixed to both ends of each sample to reduce end effects and allow uniform torque, forming a U-shaped configuration with the aid of Parafilm sealing film (Oguz et al. 2017). The midpoint of each sample was identified, and after this point, the initial buckling moment was determined at liquid pressures of 3, 6, 9, 12, and 15 mmHg, and the critical buckling rotation angle for each pressure was recorded. The configuration with no torque and the moment when the initial buckling is first barely visible is provided for each sample in Fig. 2.

### Poisson’s ratio and Young’s modulus

The specimens of the conduit materials (ePTFE, Dacron, porcine pericardium and bovine pericardium) were assumed to be homogeneous, isotropic, and exhibiting linear elastic behavior, as deformations generated due to torsional buckling are small. Therefore, Poisson’s ratio values and Young’s modulus values were computed both in axial (*x*-) and circumferential (*y*-) directions in low-strain regions, where the stress–strain curves exhibit near-linearity, along with the computation of average Young’s modulus values in both directions over the entire strain range [0, 0.1].

Linear regression was performed on a specific initial segment of the displacement-displacement data ${\Delta L}_{x} vs {\Delta L}_{y}$ obtained from uniaxial tensile tests conducted separately in the axial tension (*x*-direction) and transverse (*y*-direction) directions utilizing Eq. (1):

1 $$\nu \approx -\frac{{L}_{x}}{{L}_{y}}\frac{\Delta {L}_{y}}{\Delta {L}_{x}}$$

This equation was employed to compute the Poisson's ratio in the low-strain region for each tension direction. Equation (1) represents the first-order approximation to

2 $${\left(1+\frac{\Delta {L}_{x}}{{L}_{x}}\right)}^{-\nu }=1+\frac{\Delta {L}_{y}}{{L}_{x}}$$

for small displacements ${\Delta L}_{x} and {\Delta L}_{y}$, where Eq. (2) is the solution of

$$\nu =-\frac{d{\varepsilon }_{y}}{d{\varepsilon }_{x}}$$

with

$$d{\varepsilon }_{x}=\frac{dx}{x}, d{\varepsilon }_{y}=\frac{dy}{y}$$

being integrated from ${L}_{x}$ to ${L}_{x}+\Delta {L}_{x}$ and from ${L}_{y}$ to ${L}_{y}+\Delta {L}_{y}$, respectively. Here, ${\varepsilon }_{x}$ is the axial engineering strain, ${\varepsilon }_{y}$ is the transverse engineering strain, and ${L}_{x}$ and ${L}_{y}$ are the lengths of rectangular specimens in the axial tension and transverse directions.

Separate linear regressions were performed on the engineering stress–strain data obtained from uniaxial tensile tests conducted on each specimen taken from ePTFE, Dacron, porcine pericardium and bovine pericardium to compute Young's modulus values separately in the axial (*x*-direction) and the circumferential (*y*-direction) over the entire strain range [0, 0.1]. These individual regressions, based on Hooke's law, yielded separately fitted Young's modulus values for each orthogonal direction. The mean and standard deviation for Young’s modulus were then computed separately for each material in the *x*- and *y*-directions.

Finally, for each specimen, Young’s modulus values in both the axial (*x*-) and circumferential (*y*-) directions were computed over low-strain regions through iteration, where stress–strain curves exhibit nearly linearity. The iteration process is as follows: first, arithmetic mean Young’s modulus values computed in the *x*- and *y*- directions over the entire strain range were used as initial guesses. These guesses were employed to compute low-strain regions generated by components of the (compressive) minimum principal stress (Eq. 10) in the *x*- and *y*- directions, respectively, resulting from torsional buckling at $p=15 mmHg$ (the highest venous pressure in this study). Subsequently, these low strain regions were utilized to compute Young’s modulus values in both the x- and y- directions, where the stress–strain curves exhibit nearly linear behavior, using linear regression.

### Hyperelastic material properties

Fung’s 2D strain energy function is a widely used constitutive model to represent the stress–strain relationship of hyperelastic materials (Fung et al. 1979), given in the following form:

3 $$W=\frac{1}{2}c\left({e}^{Q}-1\right)$$

where $$Q={a}_{1}{E}_{11}^{2}+{a}_{2}{E}_{22}^{2}+2{a}_{3}{E}_{11}{E}_{22}$$.

The relationship between Green strains and stretch ratios is described by

4 $${E}_{ii}=\frac{1}{2}\left({\lambda }_{i}^{2}-1\right), i=\mathrm{1,2}$$

The Cauchy stress components can be derived as follows:

5 $$\begin{aligned}&{\sigma }_{11}=\frac{1}{2}c{\lambda }_{1}^{2}\left[{a}_{1}\left({\lambda }_{1}^{2}-1\right)+{a}_{3}\left({\lambda }_{1}^{2}-1\right)\right]\cdot \mathit{exp}\left[\frac{1}{4}\left({a}_{1}{\left({\lambda }_{1}^{2}-1\right)}^{2}+{a}_{2}{\left({\lambda }_{2}^{2}-1\right)}^{2}+2{a}_{3}\left({\lambda }_{1}^{2}-1\right)\left({\lambda }_{2}^{2}-1\right)\right)\right] \\&{\sigma }_{22}=\frac{1}{2}c{\lambda }_{2}^{2}\left[{a}_{2}\left({\lambda }_{2}^{2}-1\right)+{a}_{3}\left({\lambda }_{1}^{2}-1\right)\right]\cdot \mathit{exp}\left[\frac{1}{4}\left({a}_{1}{\left({\lambda }_{1}^{2}-1\right)}^{2}+{a}_{2}{\left({\lambda }_{2}^{2}-1\right)}^{2}+2{a}_{3}\left({\lambda }_{1}^{2}-1\right)\left({\lambda }_{2}^{2}-1\right)\right)\right]\end{aligned}$$

where $c$ [Pa], ${a}_{1}$ [-],${a}_{2}$ [-] and ${a}_{3}$ [-] are material parameters.

To facilitate nonlinear least squares curve fitting, the variables $\left\{{\lambda }_{1},{\lambda }_{2}\right\}$ were reduced to single variable ${\lambda }_{1}=\lambda$ and ${\lambda }_{2}=k\lambda$, where the value of $k$ was determined by applying linear regression to the dataset $\left(\lambda ,k\lambda \right)$.

On the other hand, experimental stress values were computed from force–displacement data using the following relations:

6 $${\sigma }_{11}^{exp}={\lambda }_{1}\frac{{F}_{11}}{{b}_{2}t}, {\sigma }_{22}^{exp}={\lambda }_{2}\frac{{F}_{22}}{{b}_{1}t}$$

where $t$ is the thickness and ${b}_{1}$ and ${b}_{2}$ denote orthogonal dimensions.

The objective function was defined as the 2-norm of the difference between the theoretical Cauchy stress ${\varvec{\sigma}}$ and the experimental stress vector ${{\varvec{\sigma}}}^{exp}$. The optimized material parameters were determined by minimizing this objective function.

### Critical buckling torque and rotation angle

The critical buckling rotation angle refers to the angle at which torsional buckling begins to occur. It is proportional to the magnitude of the torsion, also known as the critical buckling torque, applied to a structural element. For a thin-walled, isotropic, linear elastic long cylinder subjected to an end torque, the critical buckling torque can be calculated using the following formula (Flügge 1973):

7 $${T}_{cr}=\frac{\pi \sqrt{2}E}{3{\left(1-{\nu }^{2}\right)}^{3/4}}\sqrt{R{t}^{5}}$$

where $E$ and $\nu$ are Young’s modulus and Poisson’s ratio in the axial (*x*-direction) and $t$ and $R$ are the thickness and radius of the thin-walled cylinder, respectively. Equation (7) represents the critical torque required to generate buckling when the lumen pressure is zero. Based on the study by Garcia et al. (2013), a linear correlation was observed between the critical buckling torque and the lumen pressure for porcine common carotid arteries. Therefore, we propose the following equation which relates the critical buckling torque to the lumen pressure:

8 $${T}_{cr}\left(p\right)=\left(\frac{\pi \sqrt{2}E}{3{\left(1-{\nu }^{2}\right)}^{3/4}}+\beta p\right)\sqrt{R{t}^{5}}$$

where $\beta$ is a dimensionless material property representing the resistivity of a thin-walled cylinder to the torsional buckling, $p$ is the lumen pressure, and $R$ is the radius of the inflated cylinder at a given pressure $p$. The radius $R$ can be computed as a function of the pressure $p$ through the following formula for the deformation of pressure vessels:

9 $$R-{R}_{0}=\frac{p{R}^{2}}{Et}\left(1-\frac{\nu }{2}\right)$$

where ${R}_{0}$ is the radius at $p=0$. A practical predictive formulation for the buckling potential is proposed using experimental observations and available theory. Assuming that a thin-walled, isotropic, linear elastic long cylinder is subjected to an end torque (with no axial tension) and lumen pressure, a static equilibrium equation is imposed to establish a relationship between the critical buckling rotation angle and the lumen pressure. This relationship is determined by balancing the circumferential component of the (compressive) minimum principal stress with the shear stress generated by the modified critical buckling torque ${T}_{cr}\left(p\right)$ in Eq. (8), where ${T}_{cr}\left(p\right)$ linearly depends on the lumen pressure. The minimum principal stress is derived from the hoop stress due to the lumen pressure and shear stress due to torsion through the Mohr’s circle transformation:

10 $${\sigma }_{P,min}=\frac{{\sigma }_{x}+{\sigma }_{y}}{2}-\widetilde{R}$$

where

11 $${\sigma }_{x}=0{, \sigma }_{y}=\frac{PR}{t}$$

are the axial stress (no tension) and the circumferential (hoop) stress, respectively, and $\widetilde{R}$ is the radius of the Mohr’s circle given by

12 $$\widetilde{R}=\sqrt{{\left(\frac{{\sigma }_{x}-{\sigma }_{y}}{2}\right)}^{2}+{{\tau }_{xy}}^{2}}$$

here ${\tau }_{xy}$ is the shear stress occurring due to the torsion:

13 $${\tau }_{xy}=\frac{TR}{J}$$

The torque $T$ is related to the rotation angle $\varphi$ through

14 $$T=\frac{GL}{L}\varphi$$

and

15 $$J=2\pi {R}^{3}t$$

is the polar moment of inertia for a thin-walled cylinder, where

16 $$G=\frac{E}{2\left(1+\nu \right)}$$

is the shear modulus for a Hookean material. The angle ${\theta }_{P}$ between the minimum principal stress direction and the circumferential (*y*-) direction is given by

17 $$2{\theta }_{P}={\text{arctan}}\left(\frac{2{\tau }_{xy}}{{\sigma }_{x}-{\sigma }_{y}}\right)$$

Substituting Eqs. (11)–(16) in Eq. (10), we obtain the explicit equation for the compressive principal stress in terms of $\varphi , p, R, t, E, \nu , \beta$. Using Eq. (17), we set the $cos\left({\theta }_{P}\right)$ component of the compressive principal stress (Eq. 10) in the circumferential (y-) direction equal to the negative of the critical shear stress obtained by substituting Eq. (8) and Eq. (15) into (13), yields the following equation:

18 $$\left(\frac{pR}{2t}-\sqrt{\frac{{p}^{2}{R}^{2}}{4{t}^{2}}+\frac{{E}^{2}{R}^{2}}{4{L}^{2}{(1+\nu )}^{2}}{\varphi }^{2}}\right){\text{cos}}\left(\frac{{\text{arctan}}\left(\frac{2Et}{pL\left(1+\nu \right)}\varphi \right)}{2}\right)=-\frac{1}{2\pi }\left(\frac{\pi \sqrt{2}E}{3{\left(1-{\nu }^{2}\right)}^{3/4}}+\beta p\right)\sqrt{\frac{{t}^{3}}{{R}^{3}}}$$

This equation relates the critical buckling rotation angle $\varphi$ to the lumen pressure $p$, dimensions $R, t$ and mechanical properties $E, \nu$, $\beta$ of the thin-walled cylinder, where $\beta$ is anticipated to be a size-independent mechanical property.

First, tubular vascular graft samples of length 7.5 cm are chosen for the baseline case. The experimental data of the critical buckling rotation angle values of these samples at physiological venous pressures of 3, 6, 9, 12 and 15 mmHg, as measured in our current study, were utilized to test the predictive capability of the developed technique in the baseline case by fitting a nonlinear curve for determining $\beta$. This involved using the arithmetic mean of Young’s modulus $E$ and Poisson’s ratio $\nu$ values computed in the axial (x-) direction over the low-strain (linear) region (refer to Table 2) along with the arithmetic mean of thickness values for each material. The results of the nonlinear regression are presented in Table 5 under the Results section. Then, using the $\beta$ values from Table 5 and Eq. (18), critical buckling rotation angle values were computed at pressures of 3, 6, 9, 12, and 15 mmHg. Finally, ePTFE samples of length 15 cm and Dacron samples of length 15 cm and 25 cm are used to validate the performance of the technique using the same $\beta$ values for each material. Mean ± standard deviation, mean ± 2 standard deviations and relative error, which is defined as the ratio of the absolute value of the difference between the predicted critical buckling rotation angle and the experimental mean to the experimental mean, are used as performance metrics.

## Results

### Linear elastic model characteristics

#### Poisson’s ratio and Young’s modulus

**Fig. 3:**
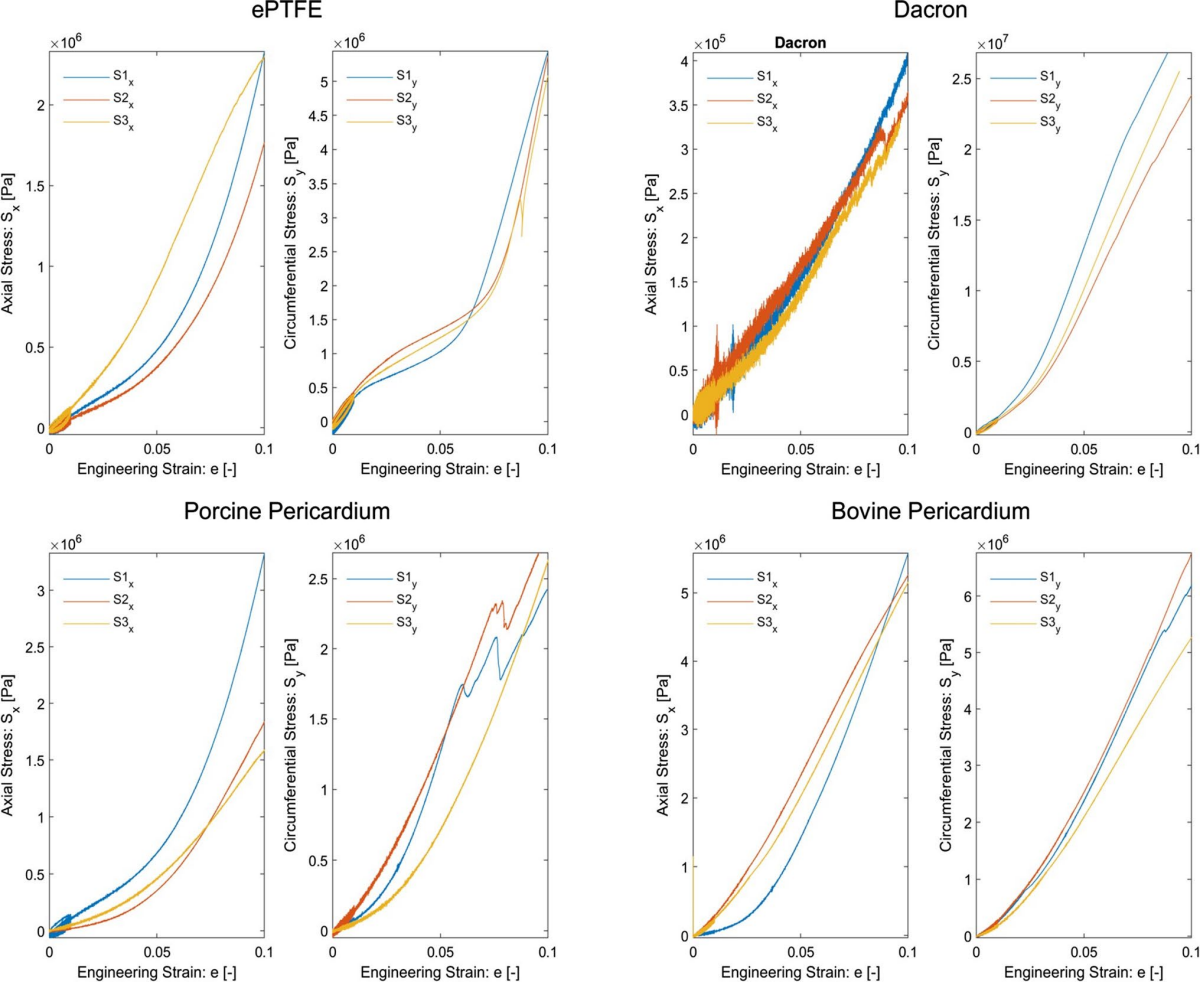
The engineering stress–strain data obtained from uniaxial tensile tests of expanded polytetrafluoroethylene (ePTFE), Dacron, porcine, and bovine pericardium are presented. For each material, experiments are performed along both the *x*-axial and *y*-circumferential directions and repeated for three samples (S1, S2, and S3) from different batches. The preconditioning protocol at the beginning is followed by a dwell step before the execution of ramp tests

The experimental engineering stress–strain data obtained from uniaxial tensile tests for ePTFE, Dacron, porcine pericardium and bovine pericardium are presented in Fig. 3.

As seen in Fig. 3, all four material types exhibit certain levels of nonlinearity in the entire strain range up to 10% elongation. However, ePTFE exhibited higher nonlinear behavior in both the axial (*x*-) and circumferential (*y*-) directions compared to Dacron, porcine pericardium and bovine pericardium, with greater curvatures of the stress–strain graphs over the 3–6% elongation ([0.03, 0.06] strain range) in the axial direction and 2–6% ([0.02, 0.06]) in the circumferential direction. All three remaining materials demonstrated lower nonlinearity in the circumferential direction, except for the fact that Dacron also exhibited lower nonlinearity in the axial direction over the entire strain range [0, 0.1]. Additionally, there is a rapid oscillation in the stress–strain data from axial tensile tests for Dacron, caused by the opening of folds on its surface.

Linear regression was implemented on both the displacement-displacement (${\Delta L}_{x}- {\Delta L}_{y})$ data and the engineering stress–strain data obtained from uniaxial tests to compute the Poisson’s ratio and Young’s modulus, respectively. The computed Poisson’s ratio and Young’s modulus values are presented in Tables 1 and 2, respectively.

**Table 1:** Poisson’s ratio values are computed from the uniaxial tensile tests in the axial (*x*-) direction and circumferential (*y*-) direction for each of the three samples of ePTFE, Dacron, porcine pericardium and bovine pericardium

	ePTFE		Porcine pericardium		Bovine pericardium		Dacron*
Sample #	Axial (–)	Circumferential (–)	Axial (–)	Circumferential (–)	Axial (–)	Circumferential (–)	Axial (–)
1	0.310	0.335	0.406	0.439	0.349	0.361	0.388
2	0.316	0.295	0.422	0.395	0.382	0.346	0.426
3	0.311	0.309	0.410	0.421	0.270	0.393	0.426
Mean $\pm$ SD	0.312 $\pm$ 0.003	0.313 $\pm$ 0.020	0.413 $\pm$ 0.008	0.419 $\pm$ 0.022	0.334 $\pm$ 0.058	0.367 $\pm$ 0.024	0.413 $\pm$ 0.022

Mean $\pm$ standard deviation (SD) Poisson’s ratio values are also determined for each direction (*x* and *y*) for each material. *Circumferential Poisson’s ratio values for Dacron specimens are not reported since the camera could not reliably track the axial movement of the fiducial markers due to the opening of folds

**Table 2:** Young’s modulus values are computed from uniaxial tensile tests in the axial (*x*-) direction and circumferential (*y*-) direction for each of the three samples of ePTFE, Dacron, porcine pericardium and bovine pericardium

	ePTFE		Dacron		Porcine pericardium		Bovine pericardium	
Sample #	Axial (MPa)	Circumferential (MPa)	Axial (MPa)	Circumferential (MPa)	Axial (MPa)	Circumferential (MPa)	Axial (MPa)	Circumferential (MPa)
1	17.11	40.17	3.73	296.17	24.44	25.40	46.07	59.33
2	13.04	36.05	3.55	226.66	14.08	28.74	52.06	62.53
3	22.55	34.23	3.22	251.34	13.42	21.81	48.89	50.36
Mean $\pm$ SD	17.56 $\pm$ 3.89	36.82 $\pm$ 2.49	3.50 $\pm$ 0.21	258.06 $\pm$ 28.77	17.31 $\pm$ 5.05	25.32 $\pm$ 2.83	49.01 $\pm$ 2.44	57.40 $\pm$ 5.15
Low-strain region: mean	9.89	31.97	2.90	115.59	7.10	16.97	24.06	35.80

Mean $\pm$ standard deviation (SD) Young’s modulus values are also determined for each direction (*x* and *y*) for each material. Additionally, axial and circumferential Young’s modulus values in low-strain regions, corresponding to the experimental loading conditions adopted in this study, are also computed, where the stress–strain curves exhibit near-linearity

Based on the arithmetic mean $\pm$ standard deviation Young’s modulus values that are computed in Table 2 in the axial (*x*-) and circumferential (*y*-) directions for each of the four materials over the entire strain range [0, 0.1], coefficient of variations ($CV$), which is defined as the standard deviation (*σ*) over the arithmetic mean (*μ*) in percentage $\upsigma \bullet 100\mathrm{\%}/\upmu$ are computed as follows:

1. ePTFE: $${CV}_{x}=22\%$$, $${CV}_{y}=7\%$$
2. Dacron: $${CV}_{x}=6\%$$, $${CV}_{y}=11\%$$
3. Porcine Pericardium: $${C}_{x}=29\%$$, $${CV}_{y}=11\%$$
4. Bovine Pericardium: $${CV}_{x}=5\%$$, $${CV}_{y}=9\%$$.

These coefficients of variation are in acceptable range (< 30%). Among these materials, Dacron has been identified as the most anisotropic, with its circumferential Young’s modulus being approximately 74 times greater than its axial Young’s modulus. On the other hand, bovine pericardium has been identified to be least anisotropic, with ${E}_{x}\approx {E}_{y}$. In addition, the low-strain regions provided in Table 3 are determined through iteration as follows:

1. ePTFE: $${\varepsilon }_{x } \in [0 0.0263]$$, $${\varepsilon }_{y } \in [0 0.0267]$$
2. Dacron: $${\varepsilon }_{x } \in [0 0.0201]$$, $${\varepsilon }_{y } \in [0 0.0206]$$
3. Porcine pericardium: $${\varepsilon }_{x } \in [0 0.0159]$$, $${\varepsilon }_{y } \in [0 0.0163]$$
4. Bovine pericardium: $${\varepsilon }_{x } \in [0 0.0185]$$, $${\varepsilon }_{y } \in [0 0.0181]$$.

**Table 3:** Statistical analysis (*p*-value) between all tested groups

	ePTFE	Dacron	Porcine pericard	Bovine pericard
ePTFE	N/A	0.39	0.5 × 10^–6^	1.2 × 10^–6^
Dacron	0.39	N/A	1.7 × 10^–7^	0.1 × 10^–6^
Porcine pericard	0.5 × 10^–6^	1.7 × 10^–7^	N/A	0.2
Bovine pericard	1.2 × 10^–6^	0.1 × 10^–6^	0.2	N/A

As expected, Young’s modulus values over the low-strain regions, where a nearly linear stress response is present, are lower than those over the entire strain range [0, 0.1].

#### Critical buckling rotation angle

**Fig. 4:**
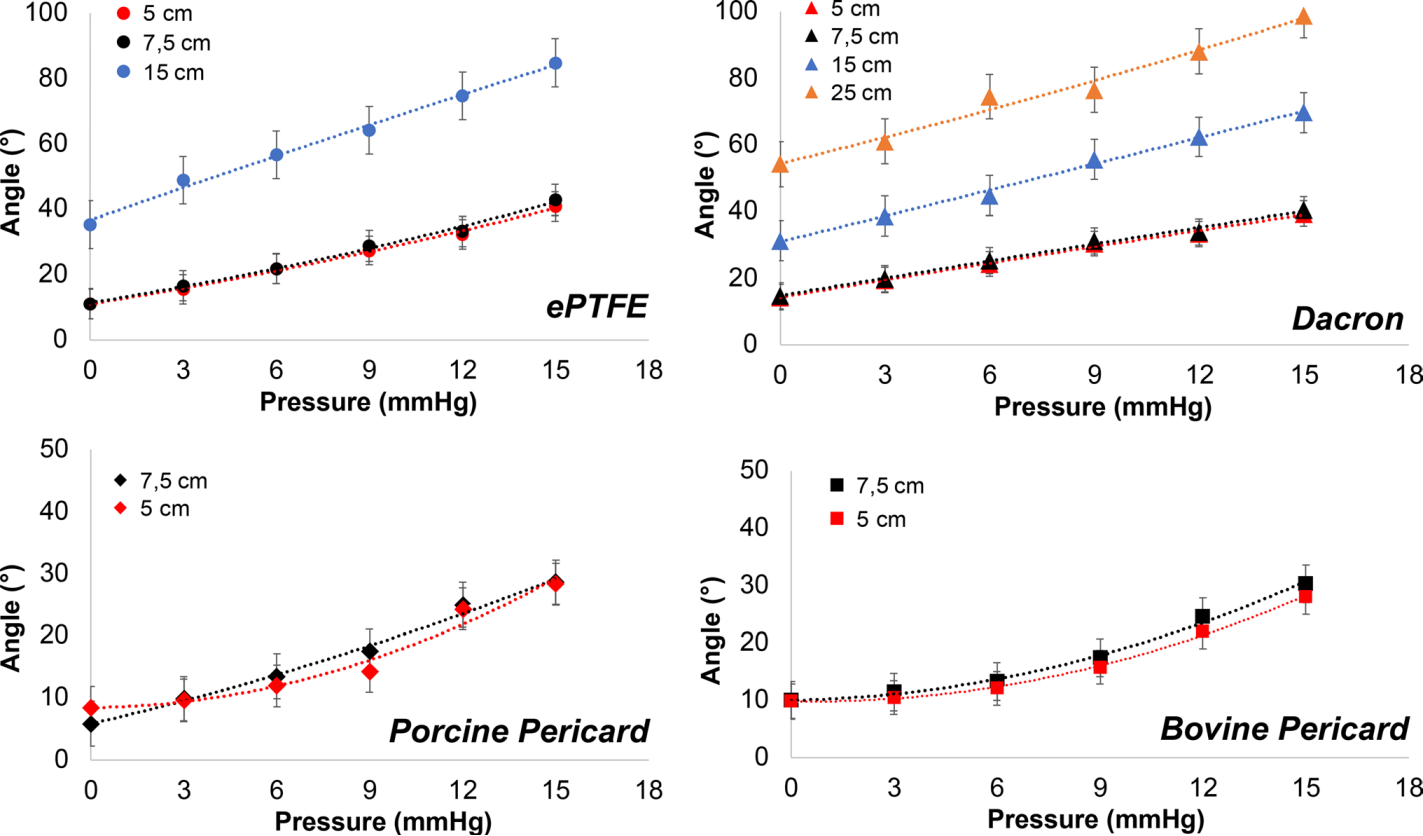
Experimentally measured critical buckling rotation angles with respect to pressure for all tested materials (ePTFE, Dacron, porcine, and bovine pericardia) at different lengths (5, 7.5, 15 and 25 cm)

The experimentally measured critical buckling rotation angle values are presented in Fig. 4.

When statistical analysis was performed, it was observed that there was no significant difference between ePTFE and Dacron (*p*-value: 0.39), between porcine and bovine pericard (*p* value: 0.2) when each tested material groups were compared with each other using *t*-test. However, there was a significant difference between all other groups (Table 3). When statistical analysis was performed, it was observed that there was no significant difference for different lengths between each sample group of materials tested based on *t*-test comparison (*p* values are 0.31, 0.36, 0.13, 0.26 between the different length of ePTFE, Dacron, porcine pericardium and bovine pericardium, respectively).

For the baseline case of this study, the developed technique was calibrated by computing critical rotation angle values at pressure levels of 3, 6, 9, 12, and 15 mmHg for tubular vascular graft samples with a length of *L* = 7.5 cm, using Eq. (18). These calculated values were then compared to the experimentally measured critical rotation values at the corresponding pressure levels, as presented in Table 4.

**Table 4:** Theoretically computed (*Theory*) critical buckling rotation angle values (in degrees) for ePTFE, Dacron, porcine pericardium and bovine pericardium tubes of length L = 7.5 cm (baseline case) are compared to the corresponding experimentally measured values (*Exp*) (in degrees) (l ± SD) with the sample size n = 4 for each material

Pressure (mmHg)	ePTFE		Dacron		Porcine pericardium		Bovine pericardium	
	Exp (deg)	Theory (deg)	Exp (deg)	Theory (deg)	Exp (deg)	Theory (deg)	Exp (deg)	Theory (deg)
3	16.6 ± 1.2	17.0	19.8 ± 2.0	15.9	9.8 ± 2.4	11.0	11.4 ± 1.3	11.8
6	21.9 ± 2.4	23.0	25.3 ± 1.7	24.2	13.5 ± 1.7	14.8	13.3 ± 1.1	15.4
9	28.8 ± 1.2	29.1	31.2 ± 2.5	32.3	17.5 ± 3.7	18.7	17.4 ± 1.9	19.2
12	33.2 ± 2.6	35.2	33.9 ± 1.4	40.4	25.0 ± 3.5	22.4	24.5 ± 2.2	22.9
15	42.8 ± 2.0	41.2	40.6 ± 2.0	48.6	28.6 ± 3.1	26.1	30.3 ± 1.8	26.6

For ePTFE and porcine pericardium, the computed critical buckling rotation angle values in Table 4 at all pressure levels of 3, 6, 9, 12, and 15 mmHg lie within one standard deviation (*σ*) of the mean. For bovine pericardium, the computed critical buckling rotation angle values at pressure levels of 3, 9, and 12 mmHg also lie within one standard deviation (*σ*) of the mean. However, at 6 and 15 mmHg, the computed critical buckling rotation values lie within two standard deviations (2*σ*) of the mean, resulting in a maximum relative error of 16%. Regarding Dacron, the computed critical buckling rotation angle values at 6 and 9 mmHg lie within one standard deviation (*σ*) of the mean, and at 3 mmHg, it lies within two standard deviations (2*σ*) of the mean. However, at 12 and 15 mmHg, the computed rotation angles fall outside of two standard deviations (2*σ*) of the mean. For Dacron, the resulting maximum relative error is 20%.

Furthermore, a direct correlation was observed between the coefficient $\beta$ and the shear modulus $G$ for each of the four materials studied in Table 5, where $\beta$ denotes the coefficient of nonlinear regression performed for critical buckling rotation angle vs. physiological venous pressure data using Eq. (18). This direct correlation means that the larger shear modulus, the higher the resistance exhibited by the material to torsional buckling under lumen pressure. This is because at a given lumen pressure, a larger $\beta$ requires a larger critical buckling torque, as indicated in Eq. (8). Based on the $\beta$ values presented in Table 5, bovine pericardium exhibits the highest resistance to torsional buckling under lumen pressure, whereas Dacron exhibits the lowest resistance, with porcine pericardium showing slightly higher resistance to torsional buckling than Dacron.

**Table 5:** The first row includes the coefficient of nonlinear regression for critical buckling rotation angle vs. physiological venous pressure data

	ePTFE	Dacron	Porcine pericardium	Bovine pericardium
$\beta (-)$	21,300	12,300	14,000	43,800
$G(MPa)$	3.77	1.03	2.51	9.02

The unitless coefficient $\beta$ represents the resistance of each material to torsional buckling. The second row includes shear modulus values for all four materials computed in the axial (*x*-) direction over low-strain regions

Finally, using the values of the (size-independent) torsional buckling resistance constants *β* for ePTFE and Dacron, namely ${\beta }_{ePTFE}=21300$ and ${\beta }_{Dacron}=12300$, respectively, from Table 5, the performance of the developed technique was validated on tubular ePTFE vascular grafts with a length of *L* = 15 cm (*n* = 3) and on tubular Dacron vascular grafts with lengths of *L* = 15 cm and *L* = 25 cm (*n* = 3 for each), having the same diameter and thickness as previous samples. Results of this validation are provided in Table 6.

**Table 6:** Theoretically computed (*Theory*) critical buckling rotation angle values (in degrees) for ePTFE tubes of length *L* = 15 cm and Dacron tubes of lengths *L* = 15 cm and 25 cm are compared to the corresponding experimentally measured values (*Exp*) (in degrees) (mean ± 2 SD) with a sample size *n* = 3 for each material and length, along with corresponding relative error values at each pressure level

Pressure (mmHg)	ePTFE (*L* = 15 cm)			Dacron (*L* = 15 cm)			Dacron (*L* = 25 cm)		
	Exp (deg)	Theory (deg)	Relative error (%)	Exp (deg)	Theory (deg)	Relative error (%)	Exp (deg)	Theory (deg)	Relative error (%)
3	48.9 ± 3.6	34.0	31	38.6 ± 7.6	31.3	19	61.0 ± 6.2	52.4	15
6	56.5 ± 3.4	46.2	19	44.8 ± 5.0	47.7	7	74.5 ± 9.0	79.5	7
9	64.2 ± 3.8	58.3	10	55.6 ± 6.8	64.2	16	76.5 ± 3.2	106.7	40
12	74.7 ± 4.6	70.5	6	62.4 ± 5.8	80.1	28	88.0 ± 5.2	133.3	52
15	84.8 ± 5.0	82.4	3	69.7 ± 6.8	96.2	38	98.8 ± 6.8	160.3	63

For ePTFE tubes of length *L* = 15 cm, the maximum relative error is calculated to be 31%, occurring at 3 mmHg pressure. The relative error decreases as the pressure increases, as evident from Table 6. On the other hand, for Dacron tubes of length *L* = 15 cm, the maximum relative error is 38%, occurring at 15 mmHg, and the relative error decreases as the pressure decreases. However, for Dacron tubes of length *L* = 25 cm, the relative error values are high at higher pressure levels, specifically at 12 mmHg and 15 mmHg.

### Fung’s 2D pseudo-strain energy function

**Fig. 5:**
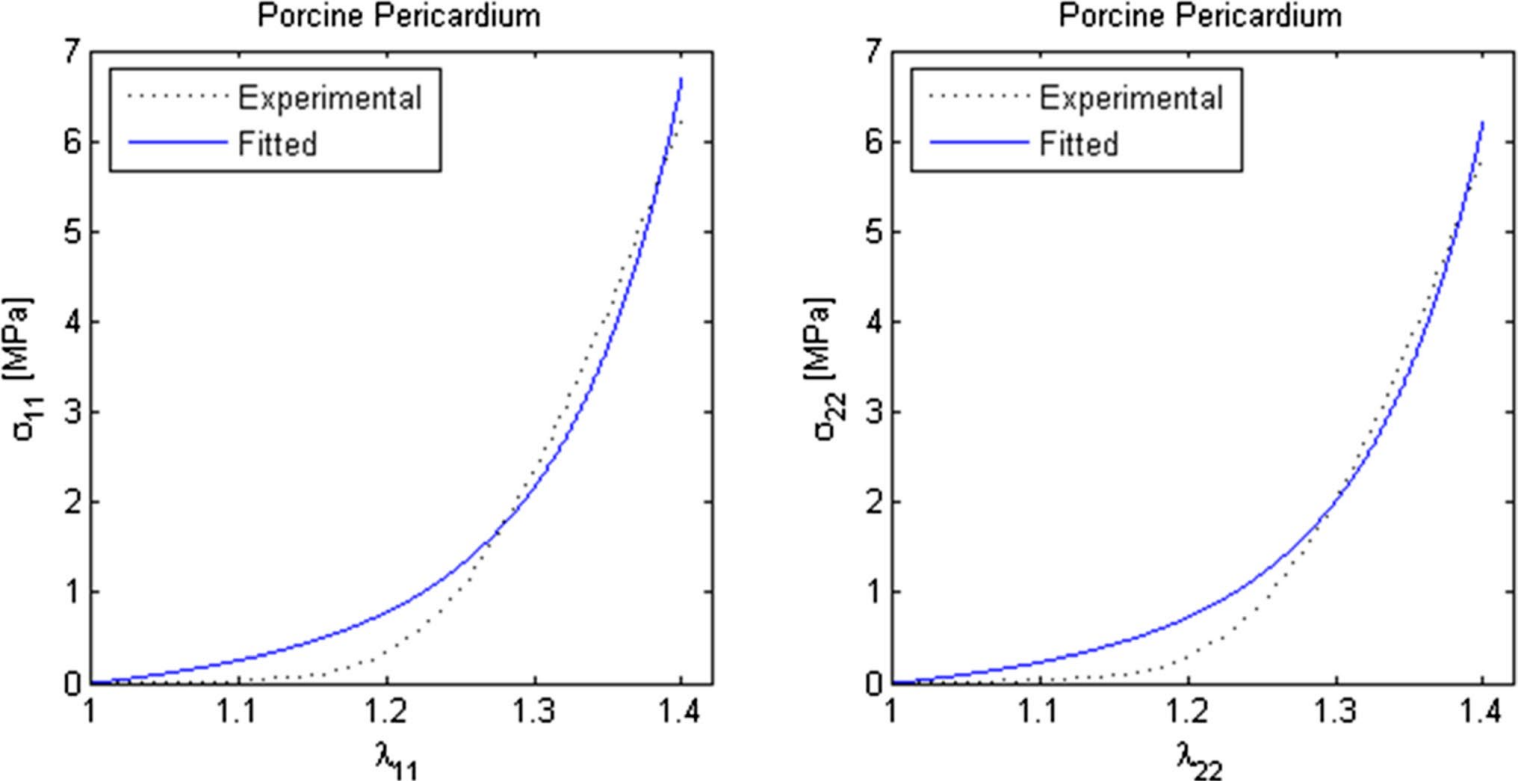
Nonlinear least-squares curve fitting is implemented to experimental stress-stretch ratio data, obtained through biaxial tensile tests, in the *x*- and *y*-directions for porcine pericardium

Fung’s 2D stress–strain curves were fitted to the stress–strain data derived from the force–displacement data obtained through biaxial tensile testing for porcine pericardium. The resulting nonlinear material parameters for porcine pericardium are presented in Table 7, along with literature values for the material parameters of the human intracranial blood vessel (Shafigh et al. 2013) for comparison (Fig. 5). Figure 5 depicts the measured data and the corresponding curves fitted to the data in both axial and circumferential directions. The figure reveals the presence of two distinct regions. The first region spans approximately 20% of the strain interval and exhibits a relatively linear behavior of the material. In contrast, the second region, which comprises higher strains (> 20%), demonstrates a noticeable transition towards nonlinear behavior. The maximum relative error in the first region is significantly higher compared to the second region due to the nonlinear nature of the strain energy density function. Notably, the data points beyond 30% strain exhibit a close fit to the nonlinear curve, with a very low error.

**Table 7:** Computed material parameters of porcine pericardium in Fung's 2D pseudo-strain energy function, and literature values of human intracranial blood vessel

Material type	c (MPa)	a_1_ (–)	a_2_ (–)	a_3_ (–)
Porcine Pericardium	2.39	0.0532	0.0000	0.7197
Human (adult) Intracranial Blood Vessel (Shafigh et al. 2013)	0.313	2.898	1.186	0.130

## Discussion and conclusions

The patency of vascular grafts is primarily associated with anastomotic intimal hyperplastic thickening (IHT) and thrombus formation. In addition to the conduit type, proximity to the suture line is reported to be the main risk factor for IHT (Loth et al. 2002), likely due to the high abnormal suture stresses involved in the anastomosis location (Ballyk et al. 1997). Mismatch of mechanical material properties (compliance) between the graft and host artery results in differential mechanical strain and can also augment intimal thickening (Salacinski et al. 2001). This case is particularly common in pediatric surgical reconstructions where vein to artery, artery/vein to an artificial conduit or pulmonary artery to systemic artery anastomoses are possible with significantly different material properties. The surgical operations intended for different vascular disease types and patient-specific properties require conduits with optimally matched material properties. The knowledge of the mechanical properties of available surgical materials is limited, but detailed consideration of characteristics able to suggest several types of conduit materials that exhibit the physiological requirements closely (Festas et al. 2019).

The large surface deformations caused by torsional buckling are a relatively overlooked mechanical condition that also creates complex strain patterns and has potential to alter the hemodynamics and biology of the anastomosis site. To the best of our knowledge, there is currently no theoretical model in the literature that correlates the critical buckling rotation angle resulting from torsional buckling to the lumen pressure. Due to the complexity of this mechanical problem and assumptions involved, an approach that is closely guided by the experimental observations is adopted here. The proposed formulation is tested against the critical buckling rotation angle measured for a variety of vascular conduit materials such as ePTFE, Dacron, porcine pericardium and bovine pericardium at a low lumen pressure. This can be generalized to any vascular conduit application with similar nonlinear elastic material properties and can find its use as a practical predictive tool. For the specific anatomy interest or surgical implantation site, the intramural pressure in conjunction with the radius, length, and thickness measurements, typically obtained from CT or MRI scans, at different blood pressure values. These values are then utilized in Eq. (18) along with the corresponding $\beta$ value provided in Table 5 to determine the critical buckling rotation angle values. As an example, two different small diameter shunts are proposed for the first-stage surgical repair of hypoplastic left heart disease; the Blalock-Taussig (BT) shunt (diameter 3.5 mm, length 35 mm) and the Sano shunt (diameter 5 mm, length 75 mm) both are made of 0.1-mm-thick ePTFE and establish a connection between the systemic and pulmonary arteries. Our formulation predicts slightly higher stability for the BT shunt compared to Sano with critical buckling angles of 130 vs. 124 degrees at 40 mmHg pressure, respectively. Although finite element simulations incorporating hyperelastic material models can provide highly accurate results, they are often computationally intensive and require significant computational resources. In clinical settings, when timely decision-making is crucial to avoid postoperative complications, the proposed approach may guide surgeons to quickly assess the buckling potential of the intended reconstruction.

Buckling estimates of emerging surgical materials not studied in this paper are also possible if their Poisson’s ratio and Young’s modulus values in the axial direction are measured for small deformations. Subsequently, critical buckling rotation angle values are measured at different lumen pressures using torsional buckling experiments like those included in this study. The $\beta$ value is then determined through nonlinear regression applied to the experimentally measured critical buckling rotation angle vs. lumen pressure dataset. Consequently, the computed $\beta$ value can be used in Eq. (18) to compute the critical buckling rotation angle for any vascular conduit made of the same material, regardless of size, i.e., radius, length and thickness, at a given lumen pressure. It is important to note that although the experimental data used correspond to the most critical low intramural pressure case, the above approach can be generalized to any physiological pressure range since uniaxial tensile tests conducted by our group covered a large enough strain range up to 10% elongation.

The possibility of buckling of grafts depends on the mechanical properties of the grafts. The earliest studies on experimental mechanical testing of biomedical conduits go back to 1979 (Hasegawa and Azuma 1979). Stress–strain curves and relaxation curves of knitted and woven grafts of both Dacron and Teflon were examined in that study using a (uniaxial) tensile testing instrument and compared with those of the canine aortic arteries. As such, Lucereau et al. (2015) investigated the relation between the tensile tests and vascular compliance in polyester textile vascular prostheses. Bustos et al. (2016) studied the mechanical characterization of a woven Dacron vascular graft. Three different models of commercially available knitted vascular prostheses were studied using longitudinal and circumferential traction tests on coated and uncoated samples.

There are a limited number of studies in the literature determining linear properties like Young’s modulus and Poisson’s ratio of ePTFE (Faturechi et al. 2019; Bouchet et al. 2019), Dacron (Faturechi et al. 2019; Coirbay 2020), porcine (Aguiari et al. 2016; Vondrášek et al. 2018; Inoue et al. 2021) and bovine pericardia (Zioupos and Barbenel 1994; Aguiari et al. 2016) through mechanical tensile tests, as these materials are examined in the framework of viscoelastic theory. However, the experimental measurements in these studies were conducted over different strain ranges, rendering direct comparisons with our values unfeasible. Thus, we conducted an expanded experimental campaign to fill this gap in the literature, spanning multiple materials and operating ranges. On the other hand, to the best of our knowledge, there are no other experimental studies in the literature except (Oguz et al. 2017), which was performed previously by our group, that measures the critical buckling rotation angle values of ePTFE, Dacron and porcine pericardium at different pressure levels. Another study by Garcia et al. (2013) reported torque vs. rotation angle measurements for the porcine carotid artery at pressure levels of 20, 40, 70 and 100 mmHg. However, the results of this study cannot be directly compared to our results since the pressure range and material properties are different.

In this study, it was demonstrated that ePTFE exhibits a highly nonlinear response across the entire strain range [0, 0.1] (or 10% elongation). Therefore, calculating Young’s modulus for a relatively large strain region of ePTFE material might not offer practical benefits. Using nonlinear material models to represent this material behavior should be preferred. However, determining the arithmetic means of Poisson’s ratio and Young’s in the axial (*x*-) direction over low-strain regions allowed us to predict critical buckling rotating angle values at physiological venous pressures of 3, 6, 9, 12, and 15 mmHg within one standard deviation of the mean since deformations corresponding to torsional buckling under the pressure of 3–15 mmHg are small (< 3% elongation). The stress–strain curves for Dacron in the axial (*x*-) direction exhibit almost linear behavior across the entire strain range [0, 0.1]. This is primarily due to the opening of folds on the surface of Dacron specimens during axial movement due to the axial extension, which makes Dacron specimens less stiff. However, Dacron specimens demonstrated very high stiffness in the circumferential (*y*-) direction, allowing us to conclude that Dacron is a highly anisotropic material. On the other hand, bovine pericardium has been identified as the least anisotropic material among all four. As a common characteristic among all four materials studied, higher stiffness in the circumferential (*y*-) direction rather than in the axial (*x*-) direction is also verified in this study.

In the literature, only a few experiments are available that investigate torsional buckling characteristics of cardiovascular conduits. This is partially due to the instable nature of the buckling mechanics which challenges the precise identification of buckling initiation. As a remedy, we consulted multiple observers, samples and recorded the critical angle while the conduits are visibly circular and just before a major collapse occurs in the circular shape. While a large range of graft lengths are tested, 5–25 cm, for porcine and bovine pericardium the availability of the tissue did not allow us to test longer samples. Still optimal sample numbers 3–4 are achieved demonstrating statistically significant conclusions and able to validate our theoretical formulation in partial fulfillment.

For vascular graft samples with a length of 7.5 cm, reliable results were obtained for ePTFE and porcine pericardium. These results consistently fell within one standard deviation (*σ*) of the mean across all pressure levels in the range of 3–15 mmHg, with increments of 3 mmHg. Results for bovine pericardium lie within *σ* of the mean at most pressure levels, except at 6 and 15 mmHg, where they lie within 2*σ* of the mean (95% confidence interval), resulting in a maximum relative error of 16%. At 6 and 15 mmHg, critical buckling rotation angle results for Dacron lie within σ of the mean, and at 3 mmHg, the computed critical buckling rotation angle lies within 2*σ* of the mean. However, at higher pressure levels, such as 12 and 15 mmHg, they fall outside of 2*σ* of the mean of the experimental data, resulting in a 20% maximum relative error.

The developed technique was validated using torsional buckling resistance constants, ${\beta }_{ePTFE}=21300$ and ${\beta }_{Dacron}=12300$, computed for ePTFE and Dacron, respectively, with samples of length *L* = 7.5 cm. It is crucial to note that these constants are size independent. The validation involved tubular ePTFE vascular grafts (*L* = 15 cm, n = 3) and tubular Dacron vascular grafts (*L* = 15 cm and *L* = 25 cm, *n* = 3 for each), with an identical diameter and thickness to previous samples. For ePTFE tubes (*L* = 15 cm), the maximum relative error was 31% at 3 mmHg, and the relative error decreased with increasing pressure. Conversely, for Dacron tubes (*L* = 15 cm), the maximum relative error recorded was 38% at 15 mmHg, and the relative error decreased with decreasing pressure. On the other hand, Dacron tubes (*L* = 25 cm) exhibited elevated relative error values at higher pressure levels (12 mmHg and 15 mmHg).

The present approach also establishes a direct correlation between the coefficient $\beta$ of the leading term of the linear polynomial ${T}_{cr}\left(p\right)$ in Eq. (8) and the shear modulus $G$. Based on this correlation, Dacron and porcine pericardium were identified as the least suitable materials for use as a vascular conduit, as they exhibit the lowest resistivity (Dacron has the lowest) to torsional buckling under lumen pressure. In contrast, bovine pericardium was identified as the most suitable material, displaying the highest resistivity to torsional buckling under lumen pressure. Therefore, the developed technique not only assists surgeons in monitoring the occurrence of torsional buckling in vascular conduits postoperatively but also enables them to select materials that are more resistant to buckling prior to surgery. In addition, a comparison of the computed material parameters of porcine pericardium with the literature values for human intracranial blood vessel in Fung's 2D pseudo-strain energy function (Shafigh et al. 2013) highlights the incompatibility of the porcine pericardium as a suitable vascular conduit. This is evident as the stress-like material parameter for porcine pericardium was found to be approximately 8 times greater than the literature value for human intracranial blood vessel.

The study is based on a large number of experimental campaigns that include uniaxial, biaxial and torsional buckling tests for ePTFE, Dacron, porcine and bovine pericardia. The number of samples used in each experiment is kept optimal as the conduits used in the tests are intended for the actual pediatric surgeries and for patients who need them with limited material availability. We minimize waste products as much as possible, leading to the inclusion of the minimum number of samples in the tests. Nonetheless, we targeted a minimum number of 4 vascular graft samples (1 sample from old experiments, 3 samples in new experiments) for each material for torsional buckling experiments and 6 rectangular samples (3 samples in the axial (*x*-) direction and 3 samples in the circumferential (*y*-) direction) for uniaxial tensile tests to obtain averages and standard deviations for statistical assessment purposes. Another limitation is the lack of availability of vascular grafts in desired sizes in the market. We had to restrict the sizes of all tubular vascular graft samples to lengths of 5.0, 7.5, 15 and 25 cm and a radius of *R* = *1 cm*. We used samples of a *L* = 7.5 cm as a baseline case for our study for which we obtained reliable results. To achieve this, we employed the formula for the critical buckling torque in Eq. (7), which is derived under the assumption of a first-order approximation for $R/L$, where the thin-walled cylinder is assumed to be sufficiently long (i.e., $R/L$< < 1). However, when torsional buckling experiments were repeated for the same shortened vascular graft samples for each of the four materials with *L* = *5.0 cm*, at higher pressure levels, computed critical buckling rotation angle values fell outside of $2\sigma$ of the mean, resulting in larger relative errors. This is mainly because the first-order approximation for $R/L$ is no longer valid, and end effects become dominant. In the validation phase of the study, the developed technique successfully predicted the critical rotation angles for both ePTFE and Dacron samples with a length of 15 cm at various pressure levels, ranging from 3 to 15 mmHg with a 3 mmHg increment. However, for Dacron samples with a length of 25 cm, the accuracy of the technique was lower at higher pressure levels. This discrepancy can be attributed to the highly anisotropic nature of Dacron samples and the challenges encountered in measuring the critical buckling rotation angle during the torsional buckling tests. Moreover, in vivo experiments to validate the mechanical characterization results of the current study are lacking. The main reason for this limitation is the lack of high-quality postoperative 4D images, especially for pediatric patients. Furthermore, mechanical characterization using these images is still a challenge, and there is no gold standard for these evaluations. However, we aim to validate our results using in vivo data in future studies. Another limitation concerns the long-term effects of the operations on the conduits and the patches. The inner layers of the conduits and patches (the layers in direct contact with blood flow) might develop plaque formation, leading to thickening and remodeling of the biomedical materials. Consequently, the mechanical behavior of these materials could undergo significant changes. This effect is not addressed in the current study, primarily due to a lack of clinical images and in vivo characterization data.

In this study, the effect of blood flow is ignored for simplicity. This important element introduces additional forces due to the complex fluid–structure interaction, where a credible numerical simulation requires advanced structural solvers that can accurately predict time-resolved large-scale deformations.

If an advanced modeling effort is justified, improved material models, such as the hyperelastic Mooney-Rivlin five parameter or Ogden third-order model can then be employed to capture the complex behavior of these materials, utilizing the stress–strain data provided here. This is particularly important for vascular grafts and natural vessels, as they can undergo significant displacements during pulsatile blood flow. It is imperative to avoid using simplified linear material properties for large displacements in the finite element method, as doing so would lead to distortions in the computational model results, deviating from the true behavior of the graft or vessel.

Torsional buckling effects of these nonlinearly behaving anisotropic materials were not fully investigated in the present analytical study due to the lack of a comprehensive theory that directly relates the critical rotation angle to blood pressure. Also, we aim for a more practical approach that will help clinical decision-making. However, as a potential future direction, the presented mathematical technique can be further developed to incorporate nonlinear material parameters and anisotropy, along with the consideration of residual strain parameters of the human cardiovascular system as introduced in our previous study (Donmazov et al. 2015). This can be achieved by utilizing noninvasive in vivo pressure versus radius, length and thickness data. Furthermore, it is straightforward to incorporate the material characteristics of diseased or aged vascular tissue, incorporating available data. For example, Roy (1881) first observed a significant decrease in the longitudinal elasticity in the aortic wall of aged population. Garcia-Herrera et al. (2016) modelled the in vivo mechanical response of ascending aortic aneurysms in individuals with Marfan syndrome through uniaxial testing. The region affected by the disease exhibited higher stress levels compared to the unaffected part of the aortic arch. Sommer et al. (2016) studied the strength of aneurysmatic and dissected human thoracic aortas via triaxial shear and uniaxial tensile testing. They found that the aortic media exhibited stronger resistance to rupture under ‘out-of-plane’ shear loadings compared to ‘in-plane’ shear loadings. Additionally, the aortic tissues demonstrated anisotropic failure properties when subjected to different shear loadings, with higher ultimate shear stresses observed in the longitudinal direction than in the circumferential direction. Vande geest et al. (2006) developed a statistical model to noninvasively estimate the distribution of wall strength distribution in aortic aneurysms from the tensile testing of surgically procured abdominal aortic aneurysm wall specimens. In conclusion, the refined technique holds the potential to analyze the three-dimensional characterization of hyperelastic surgical materials, including the assessment of residual stresses.
